# Syphilis during Pregnancy and Lactation

**Published:** 1862-01

**Authors:** Carl R. Braun

**Affiliations:** Professor of Midwifery, etc., in the Imperial Hospital, Vienna


### SYPHILIS DURING PREGNANCY AND LACTATION.

*Section* 285 *of* " *A Text-Book of Midwifery,*" *by Dr. Carl R. Braun, Professor of Midwifery, etc., in the Imperial Hospital, Vienna. Translated by* BENJAMIN LEE, A. M., M. D.

Women suffering from secondary syphilis during pregnancy usually infect the fœtus and the peripheral portions of the egg through the medium of the blood. The egg consequently perishes, and is prematurely expelled. Many cases of habitual abortion are to be explained in this manner.

Should the fœtus withstand the infectious influence of the mother until the second half of pregnancy, it then does not usually die, but is often prematurely born, giving evidence of its syphilitic heritage at birth by a puny frame, a dirty, smoky, mouldy skin; by vesicles of pemphigus on the palms of the hands and soles of the feet; by round, copperish-red spots or scars on the same surfaces; by condylomatous growths at the points of transition from cuticle to mucous membrane; and by ozena, or nasal catarrh. Such children generally die in the course of the first few days.

On examination after death, evidences of congenital or hereditary syphilis are often found, sometimes in the shape of collections of pus, of the size of a filbert, in the thymus gland, the lungs, the kidneys, or the testicles; sometimes as areolar growths in the liver. These, however, are in many cases absent. Any one who will take the trouble to examine the bodies of a number of fœtuses suspected of syphilitic infection, can readily satisfy himself of the frequency of these alterations in the various organs.

As to the connection between hereditary syphilis and the degeneration of the thymus and liver, noticed by Dubois,* I have had several opportunities of convincing myself of its reality, in company with Wedl,† Dittrich, and others. We

* Dubois, *P. Gazette Médicale,* 1850, No. 20.

† Wedl's *Pathol. Histologie,* S. 359 and 519.

usually found in the corresponding lobes of the thymus several cavities filled with a purulent fluid, or a large central cavity, containing, like the former, a turbid, yellowish fluid. This fluid did not contain the well-known gray uncleated elements which, on being treated with dilute acetic acid, exhibited the characteristic nuclei of pus-corpuscles; in the intercellular fluid, elongated threads of mucus also came into view. In the more consistent portions of the walls an exquisite variety of cellular tissue was met with.

In the liver were found cellular growths of a more definite form. In one case, a rounded tumor of the size of a bean, and tinged with yellow, projected slightly from its concave surface, with edges not distinctly defined, but shading off into a brownish-yellow, and finally, into a liver-brown color. The lighter-colored portion of the growth extended to a depth of about the third of an inch. On section, its centre was found to be intensely yellow. The consistence of the imbedded mass was generally very perceptibly denser than that of the surrounding parenchyma; to the touch it was irregularly granular; on pressure, only a small quantity of turbid fluid could be expressed. The softer parts of the new formation consisted principally of cells of the most varied forms, presenting one, two, three, or even four processes, and containing one or two nuclei of an oval form, in each of which was found one nucleolus. The nuclei were eccentric in position, and when double were often in close proximity; but in certain cases, and especially in that of the hour-glass cells, were situated at the two expanded extremities, at a considerable distance from one another.

The fusiform cells, varying greatly in their transverse diameters, were arranged upon one another, as usual, in obliquely ascending layers. The denser portions consisted mainly of fibrous fascicles. The remaining substance of the liver offered no striking anomaly.

Another phase of these cases is that in which gestation is not interrupted by the syphilitic poison; the fœtus matures and is well developed, and the child presents at first not a single symptom of syphilis. The sanitary condition of such children, however, usually presents a sad picture, when, at a later period, hereditary syphilis, scrofula, or rickets manifest themselves. Women affected with secondary syphilis, whether its evidence be confined to the skin, the mucous membrane, the iris, the bones, the cartilages, the glands, the muscles, the areolar tissue, or the internal organs; or whether its impress be stamped upon all these parts of the economy at once, abort very frequently, or give birth to dead (macerated) or miserable living children. Or if they perchance bring into the world mature and apparently healthy offspring, the hereditary disease shows itself in the course of a few days or weeks. A determination of the two following questions is therefore of great importance in practice:

1. Are the condylomata of pregnant women ever an indication of constitutional syphilis? and if so, what particular forms of them?

2. Are vaginal blennorrhœas ever an indication of venereal disease? and if so, what forms of them?

Condylomata occurring in connection with other symptoms of secondary syphilis, as for example, the syphilides, enlargement of the glands in the region of the neck, and the flexure of the elbow, ulcerations of the throat, and indurated chancres, usually, indeed, communicate the disease to the child during pregnancy. But, with regard to condylomata occurring in women who appear otherwise entirely healthy, presenting no other trace of secondary syphilis, there still exist wide differences of opinion. They are supposed by some to be specific, by others non-specific. Many number the pointed condylomata with the latter, and the broad with the former variety. It is considered by a large number of writers that they occur only secondarily, as a product of chancre, and are contagious; by another school, that they are the result of both chancre and gonorrhœa; and further, there are those who suppose that they occur primarily and without the precedence of any syphilitic symptoms, and are therefore inoculable and contagious.

The opinion that the pointed condylomata are simply the result of maceration of the epidermis or epithelium by a blennorrhagic or seborrhagic section, has its supporters, while the so-called subcutaneous or endofollicular condyloma is justly said by many to result from neither syphilis nor gonorrhœa, and hence, of course, to be non-contagious.

With so extensive a diversity of views, it becomes of the greatest importance to the gynecologist to adopt a definite opinion on the nature of the growths, in order to be able to shape accordingly his treatment of women affected with them during pregnancy, and of their children during lactation.

Simon,* of Hamburg, has published the following very important propositions with regard to them, which my own observation leads me to sustain. He says:

* Simon, Ind. spez. Path. and Therap. Red. Virchow, Bd. II, Abth. I, S. 421.

"There is, in point of fact, no reliable diagnostic sign between syphilitic and non-syphilitic warts. *The greater proportion of such growths in the anal and pudendal regions are probably of an infectious nature*, as they doubtless were before the outbreak of the venereal disease. They are hypertrophic growths of the submucous and subcutaneous cellular tissue; dry where the epidermis is thick, moist where it is thin or wanting, (*plaques muqueuses*,) and vary in form with their situation, being broad and symmetrical about the anus, and pediculated in the vagina and at its mouth."

It is extremely improbable that condylomata occurring during pregnancy in the neighborhoood of the genitals, depend simply upon the chafing of the epidermis or epithelium, for we find profuse leucorrhœa in fifty per cent. of women during the second half of pregnancy, producing redness, chafing, and even excoriation, and yet never exerting even the slightest injurious influence on the development of the fœtus, or infecting the child with gonorrhœal ophthalmia during labor, or inducing specific eruptions after birth; while, on the other hand, condyloma, in all its varied forms, is met with only in one per cent. of the women in lying-in institutions, and still more rarely among married women.

The condyloma of pregnancy must therefore be produced, not simply by a leucorrhœal secretion, but by some peculiar element of this secretion; and we may safely say that every condyloma occurring on the genitals of pregnant women justifies a suspicion of syphilis, or of some one of those hybrid poisons; and also that it may lead to symptoms of hereditary disease in the child, as has been established by my own investigations.*

Friedinger's† proposition that "children born of mothers affected with pointed condylomata (primary syphilis) are never infected by the poison," cannot be received as an axiom, because his observations extended only to the third month, while hereditary syphilis may break out even after vaccination has run its normal course, and subsequently to the limit fixed by him. As regards the relation of this subject to medical jurisprudence, the following representative dogmas of Diday,‡ and, to some extent, of Hebra, Sigmund,

* Braun, C. Zur Syphilis Congenita, Œster, Zeitsch. f. Kinderheilk. Wien. 1858. S. 201.

† Fredinger, Ebenda, S. 225, und Wiener Ges. Z., 1854, V.

‡ Diday, Traite de la Syphilis de Nouveau-nes et des Enfans a la Mammelle. Paris, 1854.

Friedinger, Wertheimber,* the author, and others, on the *transmissibility of syphilis in pregnant and nursing women to the child*, are of the greatest imporaance.

1. If the father presents symptoms of the syphilitic diathesis at the time of coition, the child will undoubtedly acquire them.

2. If the father has been infected at some previous time, but at the time of coition presents no symptoms of the disease, the child may possibly, but not certainly, escape.

3. On the same principle, the influence will certainly make itself seen if the father be in the interval between two consecutive outbreaks of constitutional syphilis, or between an attack of the primary affection and the subsequent appearance of the general symptoms at the time of connection.

4. It may also occur, if a diseased man cohabits with a healthy woman during pregnancy, without the latter showing the slightest evidence of the existence of the affection.

5. The mother implants the disease in her offspring, either by the production of an ovum, which has become diseased in consequence of the constitution vice, or if not herself infected until after conception, through the medium of her morbid blood; for if a widow, whose deceased husband was infected, contracts a marriage with a perfectly healthy man, the children by this marriage may evidence most unmistakable traces of the affection; and further, if a healthy woman suckles the syphilitic child of a stranger, her own subsequent offspring may feel the pernicious effects of her incautious kindness.

6. A mother affected with the secondary symptoms at the time of conception may transmit the disease to the child, while the father is perfectly healthy, and has never been syphilitic.

7. A woman who has taken the disease during pregnancy may transmit it to the child between the second and the seventh months; whether this be possible at an earlier or a later period is at present doubtful.

8. In a marriage where only one of the parties is syphilitic, all the children are not of necessity affected. It may even happen that only one of twins is born with the seeds of disease in its system. This appears to depend upon some preponderating influence of one or other of the parents. If both parents are syphilitic, the child is usually born with the marks of disease upon it. Hereditary syphilis begets scrofula only in the presence of certain conditions, viz: the fact that both

* Wertheimber, Ebenda, S. 145.

parents at the time of coition were in the third stage of the disease; that the constitution of the child, or of either of its parents, is lymphatic; that the child has been nourished entirely upon its mother's milk; or finally, that the anti-syphilitic treatment was not adopted at the proper time.

9. Syphilis can be communicated to the mother through the fœtus. The transmission here takes place through the blood, and corresponds to the infection of the fœtus by the father, while the mother is healthy.

10. The immediate communication of syphilis to the child (from a primary sore or virulent vaginal discharge during labor) is, in the opinion of most gynecologists and syphilographers, rare. For this there are two reasons: first, that primary sores are rarely found in the advanced stages of pregnancy; and second, that it would necessitate the prolonged contact of a particular part of the fœtus with the surface of the ulcer, or the presence of an excoriation on its skin, and the partial absence of the *ver nix caseosa.*

11. In sucklings, the infection occurs either through an infectious point on the skin of the nurse, or from the diseased milk. Generally it is first communicated from a child to the nurse, whose nipples then become the seat of condylomata, and in turn transmit it to another child.

12. Although, in the opinion of Diday, Simon, Baumes, Cullerier, and Vidal, it has been demonstrated that infection of the suckling may occur through the medium of the milk, still they allow that the affection is in such cases milder, the eruption appearing later, and often manifesting itself under the form of scrofula.

13. A woman who has given birth to a syphilitic child runs no risk of infection in nursing it, from the fact that syphilis does not occur twice as a constitutional disease in one and the same individual.

14. The passive infection of nursing women usually results from mucous tubercles in the mouth of the suckling; these being among the earliest manifestations of syphilis in the newborn, and pouring out a very abundant secretion.

15. The demonstration of the actual transmission of syphilis from the nurse to the child, in any particular case, is surrounded by difficulties. The nurse may have been cured previous to the time of the examination, and no trace of the disease be left upon her person; or if there be evidences of it, it is supposable that she may have been since infected by another child; or the child may have become diseased through the medium of the milk, although the nurse has had no visible indication of its existence in her system; or the child may have been infected by another person; or finally, a small syphilitic scar upon the nurse might easily escape the scrutiny of the physician.

16. The syphilis of the new-born is, under very favorable circumstances, transferable, although not always inoculable.

17. If the virus of a primary sore be introduced by inoculation at the same time with vaccine virus, a hyaline vesicle is formed, which ruptures on the fourth day and developes the syphilitic ulcer; the vaccine lymph, however, is vitiated, the regular pustule not forming until the eighth day, according to Sigmund, and, as Friedinger's investigations would show, not even then.

18. Vaccination, if it runs its regular course in a child affected with latent syphilis, induces a more speedy manifestation of the latter. There are cases, however, in which it does not show itself for a considerable period after vaccination.

19. Rickets, in its various grades and modifications, is a not unusual termination of hereditary syphilis, where it has not been recognized and treated as such.

20. Condylomata rarely, almost never, induce constitutional syphilis in pregnant women, and, save for their obstinacy, admit of a tolerably favorable prognosis. They are, however, extremely dangerous to the fœtus. The poison interferes with embryonal development, attacks all the most essential organs, and in the new-born enters the great mass of the circulation, without any diminution of its virulence by the action of the lymphatic system.

Physicians are still divided in their opinions as to its treatment during pregnancy, from the alleged fact that a mercurial course may kill the fœtus and induce abortion. This apprehension, however, we do not share, and to our mind the method proposed by Simon appears perfectly rational. He says: "Inasmuch as pregnant women, suffering from any well-marked syphilitic affection, usually abort in the second half of pregnancy, gestation cannot justly be considered to contra-indicate treatment; on the contrary, a judicious anti-syphilitic course may preserve the life of the fœtus. Only when confinement is very close at hand may it be more judicious to employ, for the time being, means which are merely palliative, not commencing the radical treatment until after the end of childbed.

"That mercury exerts an injurious influence on the fœtus is true only in so far as it perhaps somewhat dwarfs its growth, while syphilis, on the other hand, destroys its life."

It is not advisable to employ either decoction of sarsaparilla or iodide of potassium in place of mercury during pregnancy, the stomach and intestinal canal being too irritable for these remedies; the latter of which might, in addition, exert a very equivocal abortive tendency upon the vascular activity of the uterus.

The mildest preparations of mercury are those best adapted to the pregnant condition; externally the mercurial ointment in small quantities, and blue pill or calomel, with opium, internally. The corrosive chloride may prove poisonous to the fœtus, and cause abortion.

If a woman suffers from primary sores on the genitals during the later periods of pregnancy, an attempt must be made to heal them if possible, in order that the child may escape infection during labor. At the same time, irritating or corrosive applications should not be made, preference being given to such mild agents as lime-water, lead-water, or a decoction of cinchona combined with a little sulphate of zinc and opium, as powerful escharotics may readily induce abortion.

Condylomata, seated in the mouth of the vagina, requires during the last three months a very cautious expectant treatment. They frequently grow with rapidity in this situation, obstinately resisting every remedy, and yielding only to cauterization, by which, however, the fœtus is not in the slightest degree benefited, and premature labor may be produced. During, or soon after childbed, these growths either die of themselves, or soon disappear under the topical employment of the *aqua phagædenica* of the older writers, or some other stimulant lotion. Both ulcers and condylomata should be touched during the progress of the labor with nitrate of silver, and coated with collodion or cerate; injections of oil should be thrown into the vagina, and the child should be most scrupulously washed, especially about the neck, where the skin is thin and exposed. Every excoriation upon its surface should be touched with nitrate of silver, and any subsequent infection from the mother should be most carefully guarded against.

The mother may be permitted to nurse her child for a few weeks, should it seem requisite, provided that her affection be of a mild form, and she be not otherwise incapacitated; for if her blood were capable of building up the fœtus to maturity, her milk ought to be fit to nourish it. As a rule, however, artificial nourishment is to be preferred after the third week.

Should a mother affected with constitutional syphilis be unable to nurse her child, on which naturally rests a suspicion of syphilitic taint, it must not, even though apparently healthy, be given to another woman to nurse, inasmuch as the congenital affection often does not make its appearance until quite late, and may infect the nurse through the nipple. If, however, a nurse be found willing to run the risk of infection from her apparently healthy charge, and to undertake the business of suckling it, it becomes the duty of the physician to acquaint her with all the possible consequences that may result from such a course. The children of women affected with syphilis or condylomata must generally be artificially nourished, whether at home or in foundling hospitals, and for this purpose cow's milk answers every requirement. Only in the case of very puny children will it be necessary to keep a goat. The mother should be subjected to an anti-syphilitic treatment during lactation, in order that the taint may at the same time be eradicated from the nursling.

Inunctions of from five to ten grains of mercurial ointment on alternate days are well adapted to the treatment of the child; or, should it present actual ulcerations upon the surface, the sixth of a grain of blue mass in some mucilaginous vehicle, or still better, the third of a grain of calomel, may be administered daily until the disappearance of the affection, and then suspended for a considerable length of time, in order to avoid its more advanced results. The painful ulcers will heal very rapidly under the use of frequent applications of *aqua phagedænica*,* with opium.

If habitual abortion results from latent syphilis on the part of one of the parents, the suspected party must be subjected to a mercurial course. In subsequent pregnancies, mature and vigorous children will generally be produced as a result of this mode of treatment.—*Am. Med. Monthly.*

---

* Aq. phagedænica is a solution of chloride of calcium, holding in suspension a small amount of bichloride of mercury.

